# The precise long-term outcomes of adult IgA nephropathy by mail questionnaires: Better renal survival compared to earlier cohort studies

**DOI:** 10.1371/journal.pone.0233186

**Published:** 2020-05-15

**Authors:** Eri Imai, Joichi Usui, Shuzo Kaneko, Tetsuya Kawamura, Machi Suka, Kunihiro Yamagata

**Affiliations:** 1 Graduate School of Comprehensive Human Sciences Major of Medical Sciences, University of Tsukuba, Tsukuba, Ibaraki, Japan; 2 Department of Nephrology, Faculty of Medicine, University of Tsukuba, Tsukuba, Ibaraki, Japan; 3 Department of Public Health and Environmental Medicine, The Jikei University School of Medicine, Tokyo, Japan; International University of Health and Welfare, School of Medicine, JAPAN

## Abstract

The estimated 20-year renal survival rate of immunoglobulin A (IgA) nephropathy is approx. 60%, but it is difficult to determine the 'big picture' for IgA nephropathy because a biopsy is essential for its diagnosis. Here we attempted to determine the longer and more precise renal prognosis of IgA nephropathy. We examined 310 patients with primary IgA nephropathy. Using the patients' clinical records and histological reports from our hospital and other clinics, we surveyed their renal prognoses and treatments within 1 year post-biopsy, and we sent questionnaires to the patients who had stopped visiting any hospital. We set renal death as the primary endpoint and analyzed factors related to renal death. The total patient cohort was 267: 159 males, 108 females; average age at biopsy, 37.7 years; average estimated glomerular filtration rate (eGFR), 69.7 mL/min/1.73m^2^; urinary protein, 1.3 g/day. The mean follow-up duration was prolonged to 13.8±8.9 years (vs. 9.2±8.5 years using only medical records). The 10- and 20-year follow-up rates were 61.7% and 27.3%. The 10-, 20-year renal survival rates were 83.6% and 72.5%. Lower eGFR, hypertension, and smoking were revealed as factors independently related to renal death. To study survival of relatively benign diseases such as IgA nephropathy, longer survival rate was affected by many censoring cases. The results regarding the long-term renal prognoses of IgA nephropathy patients (including those with a mild phenotype) obtained by our analysis of a questionnaire sent to the patients provided more precise and longer-term prognoses compared to earlier studies.

## Introduction

In 1968 in France, Berger discovered and defined that immunoglobulin A (IgA) nephropathy is a proliferation of mesangial cells with IgA deposition [1]. IgA nephropathy is now understood as one of the common causes of end-stage kidney disease (ESKD) [2]. In Japan, the estimated number of individuals with IgA nephropathy is 3.9–4.5 per 100,000 people, or a total of 33,000 individuals, based on a nationwide survey by the Japanese Society of Nephrology [3]. The clinical features of IgA nephropathy are variable, ranging from independent hematuria with mild-to-moderate-range proteinuria or partly rapidly progressive glomerulonephritis. Physicians initially tended to consider IgA nephropathy as having a benign renal prognosis because of the short observation period and minimum urinary protein level in almost patients, but in 1997, Koyama et al. reported that the 10- and 20-year renal survival rates of IgA nephropathy were 85% and 61%, and thus the long-term renal outcome of IgA nephropathy was never considered to be favorable [4]. After Koyama's report about these long-term outcomes, there have been almost no similar follow-up studies worldwide.

For a determination of long-term outcomes in chronic diseases including IgA nephropathy, the difficulty in establishing through follow-up monitoring over a sufficiently long-enough period must be considered. In general, most patients with IgA nephropathy are diagnosed at a young age, and it is difficult to perform long-term follow-up on such patients because of their proclivity to change residences. If the patients' prognoses can be checked only by using medical records and the last date that patients visited a single center (i.e., a passive follow-up), the prognosis will not be accurate. Most of the previous studies surveyed only medical records or a registry, and patients who dropped out of the studies could not be followed up. In the present study, we designed a way to prolong the follow-up duration of drop-out patients. We obtained each patient's records from the referring hospital or clinic and sent a questionnaire to drop-out patients to determine the follow-up condition and the recent situation of patients with IgA nephropathy.

## Material and methods

### Study design and population

The study was based on renal biopsy records of 1,277 patients (excluding those <20 years of age) treated at University of Tsukuba Hospital from January 1985 to December 2004. Renal biopsies were performed in all patients after written informed consent was obtained. Of the 1,277 patients' records, we selected the patients with primary IgA nephropathy and excluded patients who had other complications (e.g., diabetes mellitus, an autoimmune disorder such as systemic lupus erythematosus or rheumatoid arthritis, ulcerative colitis, IgA vasculitis, hepatitis C virus infection, and hematopoietic diseases such as malignant lymphoma). We identified each patient's renal replacement therapy (hemodialysis, peritoneal dialysis, and transplantation) and treatment(s) within 1 year after the biopsy (antiplatelet drug, anticoagulant drug, oral steroid, steroid pulse, tonsillectomy, or renin-angiotensin-system [RAS]-inhibitor) from their clinical records.

We also obtained the patients' prognostic information from the referring hospitals/clinics, and we sent a questionnaire to the home of each patient who had stopped visiting our hospital or other hospitals or clinics. We made the contents of the questionnaire simple, asking only for the following information: the presence/absence of renal replacement therapy and its start date, the date of death if it had occurred; no other information about the clinical practice was requested. For patients who had undergone more than one renal biopsy, we set the start point as the day of the first biopsy. We excluded the patients whose follow-up periods were <100 days.

Histological diagnoses of the patients' renal biopsy specimens were performed at the University of Tsukuba Hospital by well-trained renal pathologists. On the subject of the adaptation of renal biopsy for IgA nephropathy, we’ve performed renal biopsies through 1980s to 2000s in the same manner, chronic nephritic syndrome with daily urinary protein level >0.5g, renal dysfunction, macrohematuria or pre-pregnancy assessment (thoughtful comments by Prof. Akio Koyama, Ibaraki Prefectural University, Ami, Ibaraki, Japan and Prof. Masaki Kobayashi, Tokyo Medical University Ibaraki Medical Center, Ami, Ibaraki, Japan). The histological findings were also graded according to the Oxford classification [5]. We could confirm pathological indicators including MESC but not T. The lesions comprised mesangial hypercellularity (M0 or M1), segmental glomerulosclerosis (S0 or S1), endocapillary hypercellularity (E0 or E1), and crescents (C0 or C1+C2). Also we excluded cases whose less than 10 glomeruli were observed. The protocol of the present study was approved by the Ethics Committee of the University of Tsukuba Hospital (No. H26-174). This study was conducted according to the Declaration of Helsinki. The medical records of the patients were anonymized for personal information so that they cannot be identified.

### Demographic and clinical data

We obtained the patients' baseline demographic and clinical characteristics from a review of their medical records taken at the time of their biopsies. We selected the age, gender, estimated glomerular filtration rate (eGFR), urinary protein (UP), body mass index (BMI), hypertension, and ex-or current-smoker at the time of biopsy as factors for analysis. Hypertension was defined as a reported history of hypertension, systolic blood pressure (SBP) >140 mmHg, or diastolic blood pressure >90 mmHg. Diabetes mellitus was defined as a reported history of diabetes or the active use of oral hypoglycemic medicine or insulin. The eGFR value was calculated by the formula for Japanese created by Matsuo et al [6]. We set renal death as the primary endpoint and analyzed factors that may be related to renal death.

### Statistical analyses

The primary endpoint was renal death, and we then analyzed the patients' outcomes by the Kaplan-Meier method. The survival differences were tested by the log-rank procedure. Factors that may be related to renal death were examined by univariate and multivariate analyses using the Cox proportional hazard model. All continuous variables are shown as mean±SD for normal distributions. Cox proportional hazards models for estimating the hazard ratios (HRs) and the 95% confidence intervals (CI) were used to identify the factors that were predictive of the development of IgA nephropathy. Clinical parameters were selected for univariate analyses. The independent variable in supplement table was assessed by analysis of variance, categorical variable was assessed by chi-square test. Statistical significance was established at p<0.05. Data were analyzed with SPSS software (ver. 24.0, SPSS, Chicago, IL, USA).

## Results

### Patients' entry and clinical characteristics

**Fig 1:**
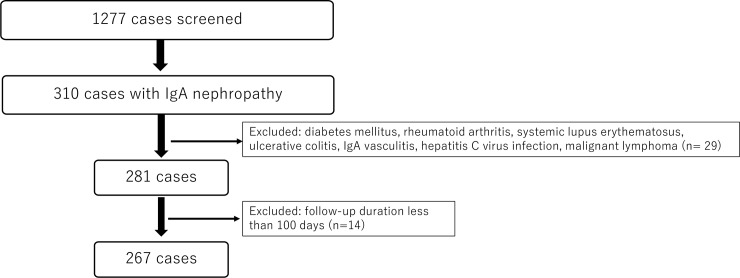
The patient’s entry.

Fig 1 is a flow diagram of the patients' study entry. Of the total of 1,277 renal pathological records, 310 patients had been diagnosed with IgA nephropathy, giving a prevalence rate of IgA nephropathy of 24.3% among all renal biopsies. After excluding the patients with secondary causes and those whose follow-up period was <100 days, we identified 267 patients. We asked about each patient's prognosis at the referring hospital/clinic and sent the above-described questionnaire to the homes of the patients who had stopped visiting any hospital.

Table 1 summarizes the clinical characteristics of our cohort (Minimal data set: S1 File). The average age of the patients at biopsy (male, n = 159 [59.6%], female, n = 108 [40.4%]) was 37.7 years old (20–77 years). The average eGFR was 69.7 mL/min.1.73m^2^ (13.0–129.5 mL/min.1.73m^2^). The average UP value was 1.3 g/day (0.1–7.5g/day). The average BMI was 23.4 (15.4–35.3). The numbers of ex-smokers and current smokers were 67 out of 165 (40.6%) (missing value was 102). Ninety of the patients had hypertension (34.7%). Within one year post-biopsy, 231 patients (86.8%) had been treated with an antiplatelet drug, 67 (25.1%) had been treated with an anticoagulant drug, 109 (40.9%) had been treated with an oral steroid, seven patients (5.3%) had been treated with steroid pulse therapy, 13 (9.8%) had undergone a tonsillectomy, and 63 (23.6%) patients had been treated with an RAS inhibitor.

**Table 1:** Clinical characteristics of participants.

	Total patients
number	267
gendar male(number, %)	159, 59.6
age (years old)	37.7±13.1
eGFR (mL/min/1.73m^2^)	69.7±25.6
Urinary protein (g/day)	1.3±1.1
History of hypertension (number, %)	90, 34.7
Body mass index	23.4±3.5
History of smoking (number,%)	67, 40.6
Anti-platelet drugs (number,%)	231, 86.8
Anti-coagulants (number,%)	67, 25.1
Oral steroid (number,%)	109, 40.9
Steroid pulse (number,%)	7, 5.3
Tonsillectomy (number,%)	13, 9.8
RAS-inhibitor (number,%)	63, 23.6
Follow-up duration without mail-survey (years)	9.2±8.5
Follow-up duration with mail-survey (years)	13.9±8.9
Renal death without mail-survey (number, %)	52, 19.4
Renal death with mail-survey (number, %)	66, 24.7

Abbreviations: eGFR; estimated glomerular filtration rate, RAS-inhibitor; renin-angiotensin system inhibitor

As the standard clinical practice at our hospital, we prescribe prednisolone 15–40 mg/day as oral steroid therapy and methylprednisolone 500–1000 mg/day intravenous drip infusion for 3 days as steroid pulse therapy.

### Patient’s histological characteristics

Table 2 summarizes the histological characteristics of our cohort (Minimal data set: S1 File). We could evaluate the histology of 201 cases. According to the Oxford classification, 43.8% of samples were classified as M1, 10.0% as E1, 29.4% as S1, 51.2% as C1+2.

**Table 2:** Histological findings of participants. (Oxford classification).

	values [number(%)]
Mesangial hypercellularity: M0/M1	113/88 (56.2/43.8%)
Endocapillary hypercellularity: E0/E1	181/20 (90.0/10.0%)
Segmental glomerulosclerosis: S0/S1	142/59 (70.6%/29.4%)
Crescents: C0/C1+C2	98/103 (48.8/51.2%)

### Long-term renal prognoses

**Fig 2:**
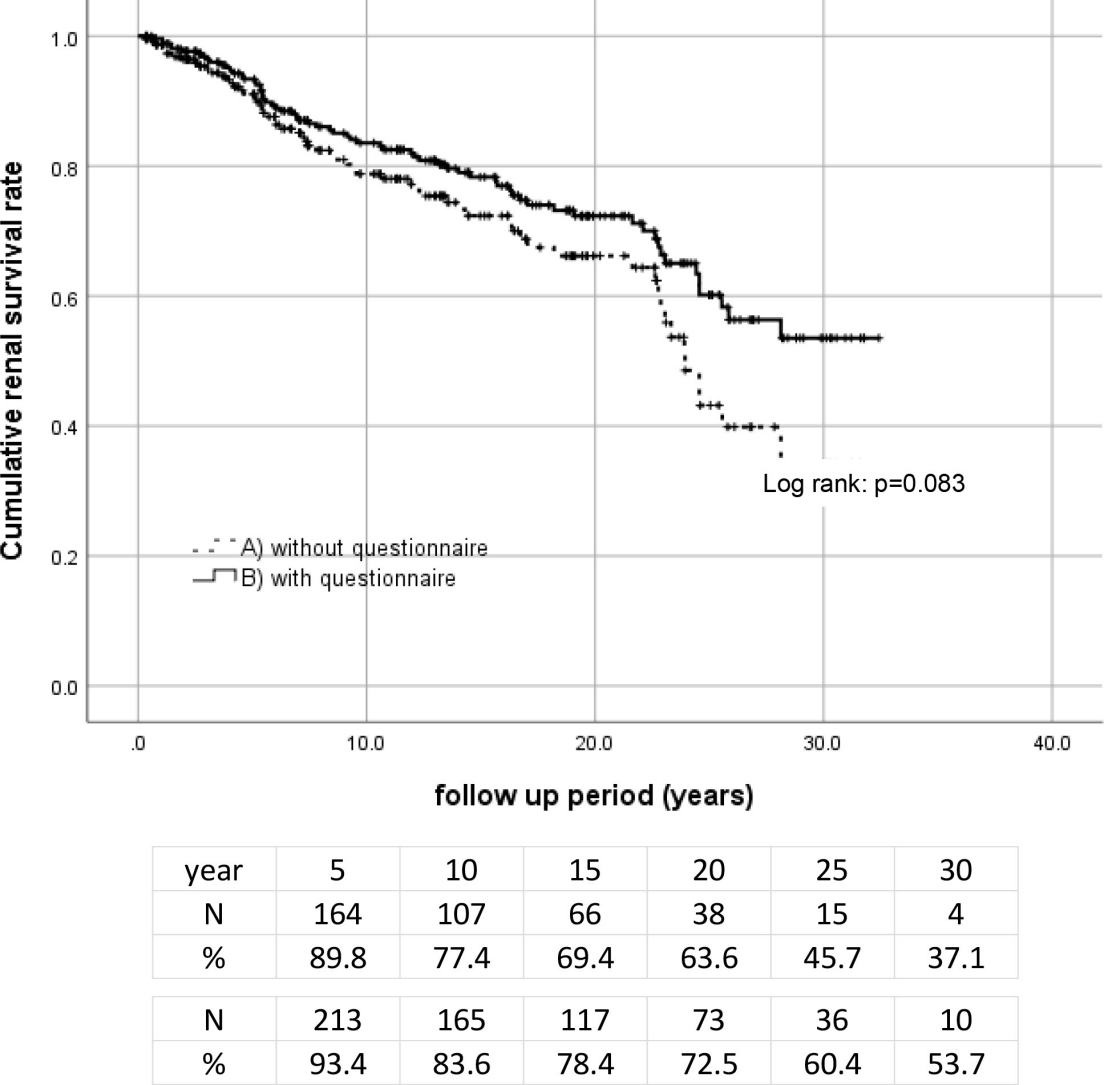
The patient’s cumulative renal survival. (A)The Kaplan-Meier curve was made using our hospital’s medical records. (B)The Kaplan-Meier curve was made from combined data adding the questionnaire results. The number of patients remaining at 5, 10, 15, and 20 years of follow-up are shown at the bottom.

The cumulative rate of renal survival of the total entries is illustrated in Fig 2 (Minimal data set: S2 File). Although the mean follow-up period obtained by our review of the patients' medical records was 9.2±8.5 years (range 0.3–31.6 years), the use of the questionnaire extended the follow-up period to 13.8±8.9 years (range 0.3–32.4 years). The follow-up rate was prolonged from 43.4% to 61.7% at 10 years and from 15.3% to 27.3% at 20 years, reducing the censored data. Based on the patients' responses to an additional questionnaire compared to a standard use of medical records, the 5-, 10-, 15-, and 20-year renal survival rates were greatly changed from 89.8% to 93.4%, 77.4% to 83.6%, 69.4% to 78.4%, and 63.6% to 72.5%, respectively, although the renal prognoses between these time points was not significant (p = 0.08).

### Univariate and multivariate analyses of the related factors associated with ESKD

We next analyzed the related factors associated with ESKD by using a Cox proportional hazards model (Table 3, Minimal data set: S1 File). We conducted a univariate analysis by age, gender, eGFR, UP, hypertension at biopsy, ex- or current smoker, and the uses of an oral steroid, antiplatelet drug, anticoagulant drug, RAS inhibitor, and pathological finding. We didn’t adopt tonsillectomy because the number of cases was small. The analysis results indicated that sex, eGFR, UP, hypertension, smoking, oral steroid, and anticoagulant drug were significant factors, and we used these factors in the multivariate analysis, which revealed that the eGFR, hypertension, and smoking were factors that were independently related to renal death. The HR for renal death was 1.0 (reference) at eGFR ≥60 mL/min/1.73m^2^, 3.33 (1.4–7.8), p = 0.06 at 45 ≤ eGFR <60 mL/min/1.73m^2^, 5.43 (2.3–12.5), p<0.001 at 30 ≤ eGFR <45 mL/min/1.73m^2^, and 18.32 (6.9–48.6), p<0.001 at eGFR <30 mL/min/1.73m^2^. And the HR for renal death was 1.9 at hypertension, 2.1 at smoking. By contrast, there was no significance for age, UP, or the use of an oral steroid.

**Table 3:** Univariate and multivariate COX regression analyses for renal death.

		Univariate			Multivariate		
	number	hazard ratio	95%CI	p value	hazard ratio	95%CI	p value
Gender							
Male	159	1.0(Reference)					
Female	108	0.6	0.4–1.0	0.06			
Age							
20–39	155	1.0(Reference)			1.0(Reference)		
40–59	92	2.08	1.3–3.5	0.04	0.79	0.4–1.4	0.33
60<	20	1.69	0.6–4.9	0.34	0.48	0.2–1.4	0.2
eGFR (mL/min/1.73m^2^)							
>60	170	1.0(Reference)			1.0 (Reference)		
45–60	39	3.64	1.7–7.7	0.01	3.33	1.4–7.8	0.06
30–45	43	9.62	5.1–18.1	<0.001	5.43	2.3–12.5	<0.001
<30	14	25.8	11.7–57.1	<0.001	18.32	6.9–48.6	<0.001
Urinary protein (g/day)							
<0.5	80	1.0 (Reference)			1.0 (Reference)		
0.5–1	70	2.13	0.9–5.0	0.085	1.24	0.5–3.2	0.65
>1	116	4.21	2.0–9.0	<0.01	1.51	0.6–3.7	0.37
History of hypertension							
no	169	1.0 (Reference)			1.0 (Reference)		
yes	90	3.2	1.9–5.3	<0.01	1.9	1.0–3.5	0.04
History of smoking							
no	98	1.0 (Reference)			1.0(Reference)		
yes	67	2.1	1.1–3.9	0.03	2.1	1.1–4.0	0.03
Oral steroid							
no	144	1.0 (Reference)			1.0(Reference)		
yes	109	1.8	1.1–2.9	0.02	1.02	0.6–1.8	0.91
RAS-inhibitor							
no	184	1.0 (Reference)					
yes	63	1.3	0.7–2.2	0.37			
Antiplatelet drug							
no	24	1.0 (Reference)					
yes	231	1.24	0.5–3.1	0.63			
Anticoagulant drug							
no	189	1.0 (Reference)			1.0 (Reference)		
yes	67	2.9	1.8–4.7	<0.01	1.34	0.8–2.3	0.3
Oxford classification							
M0	113	1.0 (Reference)					
M1	88	1.27	0.7–2.2	0.4			
E0	181	1.0 (Reference)					
E1	20	0.76	0.3–2.1	0.61			
S0	142	1.0 (Reference)					
S1	59	0.91	0.5–1.7	0.78			
C0	98	1.0 (Reference)					
C1 + C2	103	0.88	0.5–1.6	0.67			

## Discussion

We reviewed the medical records of patients with IgA nephropathy at our hospital and other hospitals and clinics, and we sent questionnaires to censored patients to determine the patients' precise renal natural history. As the result of the additional questionnaire survey, the average follow-up period was prolonged from 9.3 to 13.8 years. The 10- and 20-year renal survival rates were 83.6% and 72.5%, respectively, and were better than the rates reported in similar previous studies.

We compared various cohort studies regarding the long-term renal outcome of IgA nephropathy. The reports about the long-term prognosis of IgA nephropathy are summarized in Table 4 [4,7–14]. In 1984, D'Amico et al. reported that approx. 15% of patients reached ESKD after 10 years from the diagnosis of clinical disease, but the average follow-up period was only 5 years [7]. In 1997, Koyama et al. followed 502 patients for 11.8±6.3 years and reported renal survival rates of 85% and 61% at 10 and 20 years, respectively [4]. In 2001, Usui et al. provided the results of analyses of the long-term outcomes of an increased number of patients, but the follow-up duration was limited at 6.7 years [8]. In 2003, Geddes et al. reported their comparison of renal prognoses in four countries: the UK, Finland, Australia, and Canada [9]. The backgrounds based on clinical characteristics such as age, gender, UP, and serum creatinine differed among the four countries, and the 10-year renal survival rates in the four countries ranged from 61.6% to 95.7. By contrast, Chacko et al. reported that the 10-year renal survival rate in India was 33% [10]. This poor renal prognosis might be due to the different backgrounds of their study patients; 55% of the patients came to the clinic based on nephrotic syndrome. In that study, enrolled patients might have had progressive renal disease. Thus, the renal survival rate will differ depending on the examination system and the indications for a renal biopsy. Goto et al. obtained outcome data by adding a mail survey in 2009, but the follow-up rate and the follow-up period were insufficient [11]. In 2014, Moriyama et al. reported the long-term prognosis of 1,012 patients with IgA nephropathy in Japan [12]. They noted that approx. 50% of their population suffered renal death within 30 years. However, that study had a shorter mean follow-up of 7.9±7.1 years and a very low tracking rate at 20 years at 7.4% (75/1,012); the rate at 30 years was 1% (15/1,012). Because of the insufficient follow-up periods, the long-term renal outcome reported by Moriyama et al. cannot be precise. Although in 2012 Le et al. mentioned that 64% of a large patient group (n = 1,155) progressed to ESKD or showed a 50% decline in eGFR within 20 years, the follow-up duration in that study was too short to establish the precise renal outcomes [13]. Lee et al. reported that 70.8% of 1,364 patients progressed to ESKD within 20 years, but their follow-up rate at 20 years was only 18.5% [14]. Our present study thus has the longest follow-up duration among these previous reports, providing the most precise long-term renal outcomes in patients with IgA nephropathy.

**Table 4:** Comparison among previous reports analyzed long-term renal outcome in patients with IgA nephropathy.

Author (year)	Nation	Patient's number	No. of institutes	5-yr renal prognosis	10-yr renal prognosis	20-yr renal prognosis	follow up period	survey method	follow up rate	Follow-up period (average, years)	related factor with ESKD
D’Amico (1986)	Italy	365	3	NA	85	NA	1965–1982	by medical record	10yr 28.2% 16yr 7.9%	5	UP>1g/day
Koyama (1997)	Japan	335	52	96	85	61	1985–1993	by medical record	NA	11.8	sCr>1.4, UP>+
Usui (2001)	Japan	735	3	92	76.4	NA	1977–2001	by medical record	NA	6.7	
Geddes(2003)	UK	112	2	NA	63.9	NA	1977–1995	from published data	10yr 30.3%	7.1	
	Finland	204	1	NA	95.7	NA	1980–1995	from medical record	10yr 53.4%	10.2	
	Australia	121	NA	NA	87	NA	1959–1993	from published data	10yr 23.1%	6.1	
	Canada	274	NA	NA	61.6	NA	1963–1997	from database*1	10yr 15.0%	4.4	
Chacko (2005)	India	478	1	55	33	NA	1994–2003	by medical record	10yr 1%	5.1	HTN, nephrotic proteinuria
Goto(2009)	Japan	2283	97	NA	85	NA	1995–2005	by mail survey to hospitals	10yr 30.9%	7.3	male, <30yrs of age, HTN, UP, lower eGFR
Le (2012)	China	1,155	*2	NA	83	64	1989–2005	from database	NA	5.4	
Lee (2012)	Korea	1,364	1	NA	82	70.8	1979–2008	from database*3	10yr 39.7% 20yr 18.5%	10.2	eGFR<60, HTN, UP>1g
Moriyama (2014)	Japan	1,012	1	NA	84.3	66.6	1974–2011	by medical record	10yr 31.8% 20yr 7.4%	7.9	higher proteinuria, lower eGFR, higher UA
Present study (2019)	Japan	267	1	93.4	83.6	72.5	1985–2004	by medical record+questionnaire	10yr 61.7% 20yr 27.3%	13.8	lower eGFR, HTN, smoking

*1: the Toronto Metro Glomerulonephritis Registry, *2: Nanjing Glomerulonephritis Registry, *3: the Korean National Statistical Office and the Korea ESRD registry.

Abbreviations: NA; not available, UP; urinary protein, sCr; serum creatinine, HTN; hypertension, eGFR; estimated glomerular filtration rate, UA; uric acid.

The prognostic factors related to the long-term renal outcomes of IgA nephropathy are discussed next. D'Amico et al. reported that UP >1 g/day was a risk factor for ESKD [7]. In an investigation by Koyama et al., a serum creatinine level >1.4 mg/dl and UP>(+) were identified as ESKD risk factors [4]. Goto et al. found that male gender, age <30 years, hypertension, high UP value, and lower eGFR were risk factors for ESKD [11]. In their prospective study, Wakai et al. stated that systolic hypertension, proteinuria, hypoproteinemia, and azotemia at the initial renal biopsy would be prognostic indicators [15]. Berthoux et al. reported that hypertension, UP >1 g/day were predicting factors for ESKD [16]. In our present cohort, lower eGFR, hypertension, and smoking were independent factors related to ESKD. The UP value was significant only in the univariate analysis. UP values change over the natural course of IgA nephropathy, and thus the UP value obtained at a renal biopsy may have an unclear relationship with the long-term renal prognosis. If we set the outcome as 30% eGFR decline, but not renal death, UP might be a significant prognostic factor in multivariate analysis. In general, patients with IgA nephropathy have increasing UP and hypertension as the disease progresses, and worse renal function. Predicting factors such as lower eGFR and UP may therefore reflect only progressing disease, and of course, progressive disease means a high possibility for ESKD. We concluded that lower eGFR and hypertension were risk factors related to ESKD. In a cohort study of 971 patients with IgA nephropathy followed for 5.8 years, Yamamoto et al. reported that smoking was a significant risk factor for a 50% increase in serum creatinine [17]. Orth et al. reported that compared to patients with autosomal dominant polycystic kidney disease, in his cohort the ESKD factors were male gender and smoking [18]. Our results are similar to the findings of these previous studies, and thus smoking could be an important candidate prognostic factor for renal outcomes.

In our study, the pathological indicators regarding glomerular involvements, mesangial hypercellularity, endocapillary, segmental glomerulosclerosis and crescents weren’t independent predicting factors of ESKD in univariate COX regression analysis. There were some previous validation of Oxford classification [19][20][21][22] and they concluded these classification was helpful to predict renal outcome. However, they excluded the patients with low eGFR (less than 30 ml/min per 1.73m^2^), minimum urinary protein (less than 0.5g/day) in Oxford study [5], and their endpoint was eGFR 50% reduction. In our study, the number of patients with proteinuria<0.5g/day was 81 (30.3%), eGFR<30 ml/min per/1.73m^2^ was 43 (16.1%), different distribution with Oxford study. In addition, we observed the long follow-up natural history without aggressive therapy. As the result, we considered that these different background caused different result from previous reports. In a similar report, Alamartine mentioned that only eGFR at baseline was a predict factor, but pathologic lesions having no independent influence in the multivariate model[20]. In this report, 43 patients (23.4%) with eGFR less than 30 ml/min/per 1.73m^2^, who were excluded from the Oxford study, were entried.

Most of the previous studies seemed to survey the renal prognoses from a database based on medical records. In general, if there are more censored data, a Kaplan-Meier curve has less reliability and accuracy. Earlier investigations described many censored data; e.g., D'Amico's report mentioned that the 10-year follow-up rate was 28.3% [7]. That study may not have included milder cases of IgA nephropathy. The prior cohort studies also contained a number of drop-out patients. In 2009, to deal with the prognostic information of drop-out patients, Goto et al. used the questionnaire method for 2,283 patients treated at 97 institutes to survey the renal prognoses of IgA nephropathy [11]. Although they did not mail a questionnaire to the drop-out patients, the response rate from the hospitals was 80%. Survey methods should be considered. A questionnaire, interview methods, group surveys, telephone surveys and internet surveys are available [23]. We chose to use a questionnaire because the cost to survey was very cheap and the procedure was easier than other methods. The demerits of a questionnaire are the potential sampling error, difficulty locating patients who move, missing questionnaire responses, and a potential bias for the patients with milder cases tending to be more likely to respond. Among the questionnaire responses that we obtained herein based on three groups, i.e., medical records from our hospital (group A), medical records from the referring hospital or clinic (group B), and the questionnaire completed by patients (group C), there were no significant differences in the patients' clinical background such as age, gender, eGFR, UP, and BMI. However, the renal death rate in group A was 31.7%, that in group B was 15.4%, and that in group C was 0% (S1 Table). Although the difference among these three groups might produce study bias, we speculate that our additional questionnaire survey was able to pick up drop-out patients showing a milder phenotype. As the result of the additional mail survey, the response rate in group C was low, only 17.1%. If we could improve the response rate in group C by any means, the long-term prognosis might be better than this result.

It is also necessary to consider the temporal changes in the treatments for IgA nephropathy from the 1980s to the 2000s. In the 1980s–90s in Japan, IgA nephropathy was treated by the administration of a low-dose oral steroid. However, in some long-term cohort studies including the present study, it seems that low-dose oral steroid treatment might not influence the renal prognosis. Katafuchi et al. reported in 2003 that long-term low-dose oral steroid treatment did not improve the renal prognosis in their randomized controlled trial [24]. In our present univariate analysis, the use of an oral steroid was clearly not risk factor for EKSD. By contrast, in 2004 Pozzi et al. reported that steroid pulse treatment for 6 months significantly affected renal outcomes [25]. In recent years, high-dose steroid therapy has become the standard treatment for patients with IgA nephropathy globally, and thus the renal outcomes of the present patients who used mainly oral steroids could be similar to the natural long-term renal outcomes. In the 1990s, CKD patients with hypertension worldwide were treated with an RAS inhibitor. In 2009, Cheng et al. reported that the usage of an RAS inhibitor reduced the UP value in patients with IgA nephropathy [26]. A 2011 meta-analysis of four randomized controlled studies by Sharma et al. revealed that treatment with an RAS inhibitor improved the prognoses of nondiabetic patients with CKD at stage G1 to G3 [27]. However, we suspect that few patients in the Koyama’s study [4] were treated with an RAS inhibitor. After Koyama's work, the popularization of RAS inhibitor use might have influenced the renal prognosis during long-term follow-up. In fact, the rates of RAS inhibitor use within 1 year post-diagnosis were 23.6% in our study and 28.9% in Moriyama's report [12]. Before 2006 in Japan, an RAS inhibitor could not be administered to CKD patients without hypertension because of the Japanese medical insurance system. As a result, our univariate analysis did not show any significance regarding RAS inhibitors; perhaps hypertension is a confounding factor.

## Conclusions

We investigated the long-term renal prognoses of patients with IgA nephropathy including mild cases in part by sending questionnaires to the patients' homes. The results indicated more precise long-term prognoses compared to the values reported in previous cohort studies.

## Supporting information

S1 Table Clinical findings of each method to collect data. (XLSX)

S1 File (XLSX)

S2 File (XLSX)
